# An Affinity Propagation-Based Self-Adaptive Clustering Method for Wireless Sensor Networks

**DOI:** 10.3390/s19112579

**Published:** 2019-06-06

**Authors:** Jin Wang, Yu Gao, Kai Wang, Arun Kumar Sangaiah, Se-Jung Lim

**Affiliations:** 1 Hunan Provincial Key Laboratory of Intelligent Processing of Big Data on Transportation, School of Computer & Communication Engineering, Changsha University of Science & Technology, Changsha 410000, China; jinwang@csust.edu.cn; 2 College of Information Engineering, Yangzhou University, Yangzhou 225000, China; gaoyuyz@163.com (Y.G.); kennwong99@163.com (K.W.); 3 School of Information Science and Engineering, Fujian University of Technology, Fuzhou 350000, China; 4 School of Computing Science and Engineering, Vellore Institute of Technology (VIT), Vellore 632014, India; arunkumarsangaiah@gmail.com; 5 Liberal Arts & Convergence Studies, Honam University, Gwangju 622623624, Korea

**Keywords:** wireless sensor networks, clustering, affinity propagation, K-medoids, Internet of Things

## Abstract

A wireless sensor network (WSN) is an essential component of the Internet of Things (IoTs) for information exchange and communication between ubiquitous smart objects. Clustering techniques are widely applied to improve network performance during the routing phase for WSN. However, existing clustering methods still have some drawbacks such as uneven distribution of cluster heads (CH) and unbalanced energy consumption. Recently, much attention has been paid to intelligent clustering methods based on machine learning to solve the above issues. In this paper, an affinity propagation-based self-adaptive (APSA) clustering method is presented. The advantage of K-medoids, which is a traditional machine learning algorithm, is combined with the affinity propagation (AP) method to achieve more reasonable clustering performance. AP is firstly utilized to determine the number of CHs and to search for the optimal initial cluster centers for K-medoids. Then the modified K-medoids is utilized to form the topology of the network by iteration. The presented method effectively avoids the weakness of the traditional K-medoids in aspects of the homogeneous clustering and convergence rate. Simulation results show that the proposed algorithm outperforms some latest work such as the unequal cluster-based routing scheme for multi-level heterogeneous WSN (UCR-H), the low-energy adaptive clustering hierarchy using affinity propagation (LEACH-AP) algorithm, and the energy degree distance unequal clustering (EDDUCA) algorithm.

## 1. Introduction

The development of embedded devices as well as the micro-electro mechanical system (MEMS) wireless sensor network (WSN) as an indispensable part of the Internet of Things (IoT), has also developed rapidly in recent years [1,2,3,4,5]. WSN commonly consists of a large number of tiny sensors, which form the network in a self-organizing and multi-hop manner. WSN has its unique features such as easy deployment, self-organization, low cost and fault tolerance, etc. Therefore, it has been widely used in many applications such as environmental detection [6], industrial production monitoring [7] and smart home [8].

One of the key research issues for WSN is energy efficiency [9,10,11,12,13,14], since the tiny sensors are generally powered by limited battery supply, and the battery replacement for these sensors is impossible because of the enormous quantity and harsh environment. Therefore, it is necessary to design energy-efficient routing protocols. In clustering-based protocols, all sensors are divided into different clusters according to some specific rules. Usually, one cluster head (CH) is selected in each cluster and the other nodes will communicate with the CH directly. By introducing clustering methods, the following benefits can be achieved. First, the clustering technique makes the data transmission between sensors easy and the network topology is easy to organize. CH can adopt the Time Division Multiple Access (TDMA) schema to its cluster members for data uploading. In this way, package loss rate will be reduced and much energy can be saved from data retransmission. Second, clustering can help to alleviate the hot spots problem which is caused by centralized data transmission. The rotation of CHs can largely balance the energy consumption of different sensors. Third, clustering can reduce the total energy consumption of the network by reducing the average intra-cluster communication distance.

Although many benefits can be obtained by introducing clustering methods, some drawbacks still exist in the practical applications. Many clustering protocols such as low-energy adaptive clustering hierarchy (LEACH) [15] and power-efficient gathering in sensor information system (PEGASIS) [16] prefer to select the CHs in a random way, which causes the uneven distribution of CHs. Sensors have to adopt long distance communication with CHs in those areas which contain few CHs. One good solution for this problem is to set the competition range of sensors during the selection procedure. Once a sensor claims to be a final CH, other sensors in its competition range will give up the election to avoid the dense distribution. Many schemas are less energy efficient and the total energy consumption of the network is high, because they do not optimize the intra-cluster and inter-cluster communication distance. Therefore, it is a good choice to introduce a heuristic algorithm such as ant colony optimization (ACO) and particle swarm optimization (PSO) to decrease the average communication distance. Additionally, premature death of sensors occurs in some protocols. Those protocols commonly not take the residual energy into consideration when they select the CHs. Premature death of CH will result in blind spots for monitoring and the performance of the network will sharply decrease. Therefore, it is important to take energy balancing strategy in routing protocol designing.

In order to solve the above problems, affinity propagation (AP) and the modified K-medoids [17] are combined for better clustering in this paper. AP is commonly used to calculate the similarity of nodes. It adopts the similarity matrix to represents the similarity between different nodes and the value of the diagonal of the similarity matrix is used as a criterion to judge whether the node can become a cluster center. One important function of AP is that it can figure out the optimal number of clusters according to the distribution of nodes. K-medoids, which is a machine learning based clustering method, is modified by taking the residual energy into consideration. By combining the modified K-medoids with AP, clustering will not be limited to the number of clusters and the initial cluster centers. In this paper, the AP algorithm is firstly introduced to calculate the optimal number of clusters and the initial cluster centers. Then, K-medoids is adopted to form the final clustering results by iteration based on the initial cluster centers. By adopting the elaborate selected initial cluster centers, the proposed method select more reasonable CHs compared to the traditional K-medoids algorithm.

The rest of this paper is organized as follows. In Section 2, some classic routing protocols which adopt clustering techniques are discussed. In Section 3, the system and energy models are given. Section 4 illustrates the proposed algorithm in detail. Section 5 provides extensive simulation results with analysis and comparison. Some discussions are presented in Section 6, and Section 7 concludes this paper.

## 2. Related Work

Some classic or recent literature about the clustering techniques for WSN is listed in Table 1. Low Energy Adaptive Clustering Hierarchy (LEACH) [15] is a hierarchical routing protocol with two layers, CHs layer and member layer. In each round, every sensor generates a random number and once the number exceeds the threshold value, it will be elected as a CH. One of the serious weakness of LEACH is that the selected CHs are uncontrolled and they distribute unevenly among the sensor field. Additionally, it does not consider the residual energy of sensors which may lead to the premature death of sensors.

LEACH-centralized (LEACH-C) [18] is an improved version of LEACH. In LEACH-C, before CHs selection in each round, the information of sensors such as residual energy and position are reported to the base station (BS). The BS calculates the average residual energy of the network and excludes the weak sensors from the candidate CHs. The main drawback of LEACH-C is that the topology of the network is not optimal and the total energy consume of the network is high in each round.

LEACH using the AP algorithm (LEACH-AP) [19] adopts iterations to select CHs. The proposed scheme generates clusters by iteration to exchange information between sensors. However, the number of clusters still needs to be set manually.

Power-Efficient Gathering in Sensor Information Systems (PEGASIS) [16] is a chain-based clustering algorithm. The whole sensors in the network are connected into several chains and the chain leaders are dynamically selected. Chains are constructed by greedy algorithm and each node in the chain takes turn to be the leader. Each node on the chain only needs to communicate with its nearest neighbor. Due to the chain construct of the network, once a sensor in the chain fails, the whole chain will stop work. Additionally, chains in a larger-scale network will result in severe network latency.

Hybrid energy-efficient distributed Clustering (HEED) [20] is another hierarchical routing protocol. The mainly contribution of HEED is that it firstly presents a novel method for CHs selection by competition. The residual energy of sensors is taken into consideration for CHs selection. Each node calculates its average minimum reachability power (AMRP) to determine which CHs should join. 

The threshold-sensitive energy-efficient sensor network protocol (TEEN) [21] is an event-driven and responsive algorithm. During the process of CHs selection, each candidate CH broadcasts two parameters, soft threshold and hard threshold, to other sensors among the cluster. The hard threshold records data eigenvalues, and the soft threshold records the maximal range of data change. One of the shortcomings of TEEN is that it achieves the clustering result by iterating and the algorithm is executed in the local sensors with low computing power. Therefore, it leads to great network latency.

The saving energy clustering algorithm (SECA) [22] is a centralized clustering algorithm. Candidate CHs are firstly selected according to the average residual energy. Then, the modified K-means algorithm which considers the location and residual energy is adopted to determine the final CHs. The algorithm mainly contains two parts, set-up phase and steady-state phase. In the set-up phase, the center location is calculated to set the initial means of points according to the location of the stationary nodes. Then K-means algorithm is utilized to divided the network into several clusters according to the previous initial means. The steady-state phase mainly conducts the data transmission. Member nodes send data to their corresponding CHs using the allocated TDMA schema and the CHs forward the data to the sink.

Energy aware unequal clustering (EAUC) [23] is a fuzzy logic-based clustering algorithm. In EAUC the sensor field is divided into several heterogeneous clusters. Those clusters close to the sink own smaller scale. The fuzzy logic system is utilized for CHs selection in EAUC and it comprehensively considers the features of sensors such as residual energy, position and the number of neighbors. The output of the fuzzy logic system is the possibility for a sensor node to be select as a CH.

The energy-efficient unequal clustering (EEUC) [24] Mechanism is an unequal clustering algorithm. In EEUC, the size of each cluster is calculated by the distance between its corresponding CH and the sink. CHs are elected via competition and the competition range enlarges with distance increasing. Using this method, clusters close to the sink generally own less members and the energy used for intracluster communication is reduced.

Unequal cluster-based routing scheme for multi-level heterogeneous WSN (UCR-H) [25] is a multiple CH-based clustering algorithm. The size of the cluster in UCR-H is contrary to EEUC. Clusters close to the sink own more sensor nodes. Meanwhile, multiple CHs are selected in one cluster to ease the burden of forwarding. The optimal number of clusters is calculated by linear programming.

The energy degree distance unequal clustering algorithm (EDDUCA) [26] partitions the network using Sierpinski Triangle. The triangle of the outer of the sensor field generally contains more sensor nodes.

## 3. System Model

### 3.1. Network Model

**Figure 1:**
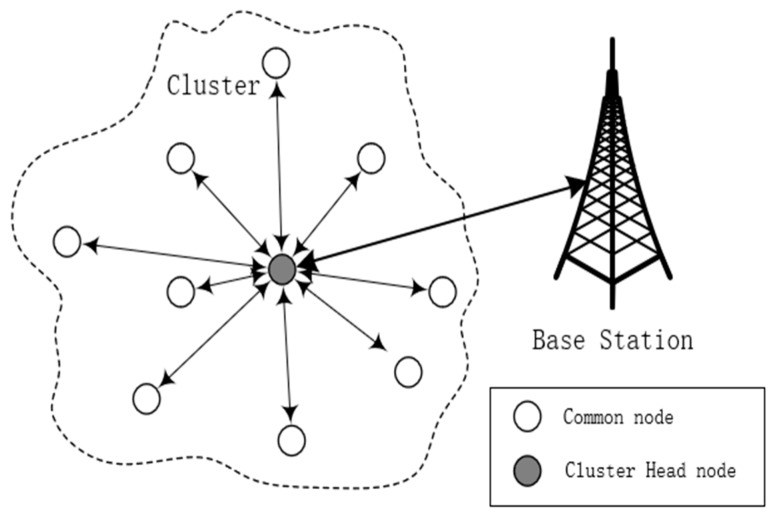
Network model.

In this paper, the network is composed of numerous sensors as well as a BS, as shown in Figure 1. Some physical information of the sensor field such as temperature and humidity are detected by sensors and they transmit their monitored data to its corresponding CHs. There are two types of roles for the sensors to play. Member nodes need to monitor the surroundings and send the monitored data to corresponding CHs. CHs not only need to detect the information of the environment, but also need to receive the data packages from their members and conduct data fusion. Finally, the fused data is uploaded to the BS by the CHs. The following assumption are made to conduct the simulation conveniently.

1. All the sensors are deployed in a rectangle area by planes or other vehicles and they keep stationary after they are deployed.
2. Sensor nodes can be identified by their unique ID.
3. Each sensor owns the knowledge of its position by the equipment such as the Global Positioning System (GPS), and they can get the information of other nodes by information exchange.
4. All the sensors own the same initial energy and their batteries cannot be changed. Once they exhaust their energy, they will be useless.

### 3.2. Energy Model

**Figure 2:**
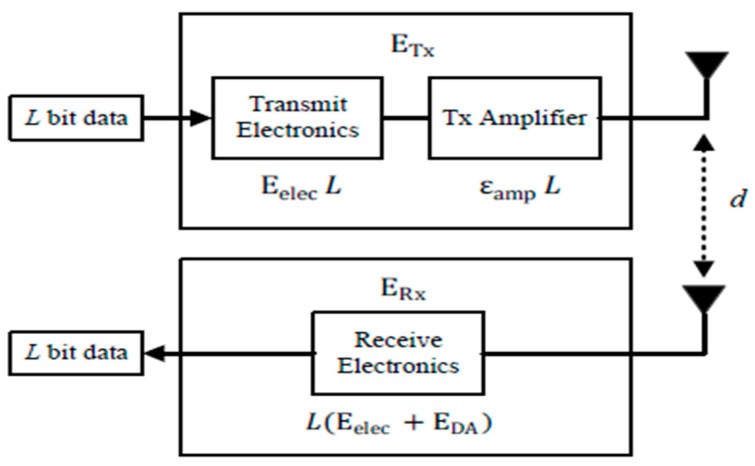
Network model.

As the research [15,27,28,29,30] has discussed, the energy used for transmission accounts for the majority of the total energy consumption. Therefore, energy consumption used for transmission is only considered in this paper. The energy used for transmission is generally divided into two parts, sending and receiving units, as shown in Figure 2. In the sending unit, the digital signal is transformed into an analog signal by the transmit electronics and then the analog signal is strengthened by the amplifier. The power of the amplifier is adjustable and it uses different power according to the communication distance. A threshold value $d_{0}$ is calculated to adjust the power of amplifier. If the communication distance exceeds the threshold value $d_{0}$, free space model is used, otherwise, a multi-path fading model is used. In the receiving unit, the analog signal is transformed into digital signal again and the energy used in this part only depends on the amount of data.

The total energy $E_{Tx}$ used for sending unit can be calculated using the Formula (1).

(1) $$E_{Tx}\left(L,d\right)=E_{elec}\cdot L+\varepsilon_{amp}\cdot L$$

where *d* represents the communication distance between the source node and the target node. *L* denotes the length of data package. $E_{elec}$ represents the energy consumed by transmitting one-bit data between two sensors. $\varepsilon_{amp}$ is the energy consumption for the amplifier and it can be calculated by Formula (2).

(2) $$\varepsilon_{amp}=\{\begin{array}{c} \varepsilon_{fs}\cdot d^{2},\;when\;d\leq d_{0}\\ \varepsilon_{mp}\cdot d^{4},\;when\;d>d_{0} \end{array}$$

where $\varepsilon_{fs}$ represents the energy consumption for free space model and $\varepsilon_{mp}$ represents the energy consumption for multi-path fading model. Additionally, $d_{0}$ is the threshold value for amplifier to adjust its power. $d_{0}$ can be calculated by Formula (3).

(3) $$d_{0}=\;\sqrt{\frac{\varepsilon_{fs}}{\varepsilon_{mp}}}$$

The total energy $E_{Rx}$ used for receiving unit can be calculated using the Formula (4).

(4) $$E_{Rx}\left(L\right)=E_{elec}\cdot L$$

## 4. The Proposed Affinity Propagation-Based Self-Adaptive (APSA) Algorithm

In this section, a detailed illustration of the affinity propagation-based self-adaptive (APSA) algorithm will be given. Initial phase, set-up phase and communication phase are contained in APSA. During the initial phase, sensors obtains the necessary information from their neighbors for network forming. After all the preparations are finished, set-up phase will start. In set-up phase, the network topology is determined by AP and the modified K-medoids. Then the network enters into the communication phase and data transmission is conducted in this phase. 

### 4.1. Initial Phase

After all the sensors are deployed, the system begins to enter the initial phase. In the initial phase, the network has not been organized and sensors can only get their own location by GPS and record the information of residual energy. Then sensors begin to exchange their own information with their neighbors until the sink obtains the information of all the sensors. When the information exchange is finished, the system enters set-up phase.

### 4.2. Set-Up Phase

The main goal of the set-up process is to find the CHs and divide all the sensor nodes into appropriate clusters. During this phase, the AP algorithm is firstly introduced to find out the optimal cluster number and the position of initial cluster centers. Then K-medoids algorithm is used to achieve the final clustering result. In the traditional K-medoids algorithm, the initial cluster centers are randomly selected which means that the algorithm needs to iterate more time to converge. Additionally, the traditional K-medoids runs easily into local optimal solutions. With the purpose of solving the mentioned problems, AP is adopted to figure out the initial cluster centers to enhance the performance of K-medoids.

Firstly, the similarity between sensors can be calculated using the following formula:

(5) $$s\left(m,n\right)=-\|X_{m}-X_{n}{\|}^{2}\;m,n\in \left\{1,\ldots ,N\right\},m\neq n$$

where X represents the location of sensors and s(m,n) denotes the similarity between node m and node n which is calculated by the square of their Euclidean distance. The similarity indicates whether the node n is suitable to be the CH for node m. For each node n, a real number s(n,n) represents the preference that it will be chosen as a cluster head node. s(n,n) is calculated by Formula (6).

(6) s(n,n)=p

where p represents the negative cost of adding a cluster. By numerous simulations, when p is set as –6000, the AP algorithm can achieve a good result.

r represents the responsibility and a represents the availability. a is firstly set as zero, and then r and a can be updated using Formulas (7) and (8).

(7) $$r\left(m,n\right)=s\left(m,n\right)-\underset{n^{\prime }\neq n}{\max}\left\{s\left(m,n^{\prime }\right)+a\left(m,n^{\prime }\right)\right\}$$

(8) $$a\left(m,n\right)=\{\begin{array}{ll} \underset{m^{\prime }\neq m}{\sum }max\left\{0,r\left(m^{\prime },n\right)\right\} & if\;n=m\\ min\left\{0,r\left(n,n\right)+\underset{m^{\prime }\notin \left\{m,n\right\}}{\sum }max\left\{0,r\left(m^{\prime },n\right)\right\}\right\} & if\;n\neq m^{\prime } \end{array}$$

where r(m,n) is defined as the value of the degree of node n if node n is selected as the CH of node m. a(m,n) represents the appropriate degree of node m to select n as its CH. Finally, Formula (9) is used to calculate the initial cluster centers.

(9) $$T=arg\underset{n}{max}\left\{a\left(m,n\right)+r\left(m,n\right)\right\}$$

where T represents the set of the initial cluster centers. The pseudocode of AP is described as Algorithm 1.

**Algorithm 1**: The method for obtaining initial cluster centers**Input:** the coordinate set of *N* sensor nodes $\left\{X_{1},X_{2},X_{3},\cdots ,X_{N}\right\}$; **for***i* = 1, 2, 3, …, *N***do**  **for**
*j* = 1, 2, 3, …, *N*
**do**   **if**
*i* == *j*
**then**    set *preference*
$S_{i,i}=-6000$
    **else**     calculate *similarity*
$S_{i,j}=-\|X_{i}-X_{j}{\|}^{2}$
    **end if**  **end for** **end for**
**Repeat**
 **for**
*i* = 1, 2, 3, …, *N*  **for**
*j* = 1, 2, 3, …, *N*   calculate *responsibility*
$R_{i,j}=S_{i,j}-\underset{j^{\prime }\neq j}{\max}\left\{S_{i,j^{\prime }}+A_{i,j^{\prime }}\right\}$
   **if**
*i == j*
**then**    $A_{i,j}=\underset{i^{\prime }\neq i}{\sum }max\left\{0,R_{i^{\prime },j}\right\}$   **else**    $A_{i,j}=min\left\{0,R_{j,j}+\underset{i^{\prime }\in \left\{i,j\right\}}{\sum }max\left\{0,R_{i^{\prime },j}\right\}\right\}$   **end if**  calculate $T=arg\underset{j}{max}\left\{A_{i,j}+R_{i,j}\right\}$  **End for** **End for****Until***T* does not change

The initial cluster centers obtained through the AP algorithm are not optimal, and there may be outliers. Due to the disadvantages above, the K-medoids algorithm is adopted to further optimize the clustering results. K-medoids adopts real points as the cluster centers instead of virtual points, and therefore the absolute errors can be effectively reduced. By combining the advantages of AP and K-medoids algorithm, the distance between the member node and its corresponding CH is minimized. Formula (10) describes the problem that the algorithm needs to solve. We want to study how to minimize the criterion of the absolute error σ.

(10) $$\sigma =\overset{k}{\underset{i=1}{\sum }}\underset{s\in T_{i}}{\sum }dist\left(s,T_{i}\right)$$

where s is the common node in $T_{i}$ and $T_{i}$ represents the set of nodes of cluster i. In order to minimize the criterion of the absolute error σ, greedy method is adopted to achieve this object.

A set of nodes of a cluster is represented as $T=\left\{\tau_{1},\tau_{2},\ldots ,\tau_{j},\ldots ,\tau_{k-1},\tau_{k}\right\}$. Then a node $\tau_{random}$ is randomly selected in the network to replace the node in set T, meanwhile, the residual energy of $\tau_{random}$ which is randomly selected must be richer than other nodes in set T. Formula (11) describes the replacement method.

(11) $$T^{\left(t+1\right)}=\{\begin{array}{ll} T^{*} & ,\sigma^{*}-\sigma^{\left(t\right)}<0\\ T^{\left(t\right)} & ,otherwise \end{array}$$

where $T^{*}=\left\{\tau_{1},\tau_{2},\ldots ,\tau_{random},\ldots ,\tau_{k-1},\tau_{k}\right\}$. Then, the network is temporarily reclassified into k clusters. The new absolute error criterion $\sigma^{*}$ can be calculated by Formula (10). Compared with original $\sigma^{\left(t\right)}$ in the *t-*th time iteration, if $\sigma^{\left(t\right)}$ is greater than $\sigma^{*}$, $T^{\left(t+1\right)}$ will be replaced by $T^{*}$. 

In the process of iteration, we focus on the remaining energy of each CH. In each iteration, once the average residual energy of all sensor nodes is greater than that of a CH, the CH must give up the election and become a member node. By repeating Formula (11), the final clustering results can be obtained. The pseudocode of the modified K-medoids is described as Algorithm 2.

**Algorithm 2**: The method for clusteringlet T as the set of initial cluster centers;calculate the number of initial cluster centers k=‖T‖

**Repeat**
 assign each remaining common node to the cluster with the nearest medoid; randomly select a common sensor node $\tau_{random}$; calculate the cost function $S\left(S=\sigma^{*}-\sigma \right)$ of swapping node $\tau_{j}$ with $\tau_{random}$; **if**
*S<0*
**then**  swap $\tau_{j}$ with $\tau_{random}$ to form the new set of *k* clusters; **Until** no change**Output**: a set of *k* clusters.

### 4.3. Communication Phase

The clustering algorithm is executed in the remote server and the result of the clustering is sent to each sensor by broadcasting. When sensor nodes receive the clustering message, the real network architecture is established. In each round, the member nodes communicate with their corresponding CHs to upload the monitored data and their own residual energy. Each CH gather the monitored data of their members and then data fusion is conducted to filter the redundant data. Next, the compressed data is transmitted to the BS. At the end of each round, the BS uploads all the data of this round to the remote server. Finally, the remote server will quickly calculate the topology of next round of the network and return it to the BS. The BS determines whether it is necessary to send the reconstructed message by comparing whether the topology information of the previous round and current round are consistent. The next round starts with a message from BS and the network repeats the process from the set-up phase.

## 5. Performance Evaluation

### 5.1. Simulation Parameters

Matlab as a powerful project software has been widely used in automatic control, machine design and mathematical statistics. Researchers can solve the complicated engineering problem efficiently using the integrated toolbox in Matlab. Additionally, Matlab can dynamically simulate operation of the system and conveniently visualize the data. Matlab is run with version of R2016a in a personal computer equipped with an Intel Core I5 central processing unit (CPU) to test the performance of the proposed APSA. The simulator randomly generates the sensors in a specific area with the same initial energy. A round is used as the period of the network and in each round, a sensor needs to upload a data package to the base station via single or multi-hop communication. According to [19], considering the discriminability and run time of the simulation results, the initial energy $E_{init}$ of each node is set as 2J and the data aggregative energy $E_{DA}$ is 5nJ/bit/signal. All the relevant parameters used in the simulation are listed in Table 2.

An assumption is made that the sensors can communicate with the other nodes in their transmission range. In each round, each node generates a data package which contains the monitored information of surroundings and the target of the network is to gather all the packages of sensors. In the simulation, 50 sensor nodes are firstly deployed in a $100\times 100\;m^{2}$ sensor field in a random way. Then a BS is set at the center of the monitoring area. The AP algorithm is executed to search the optimal initial cluster centers and adopt the modified K-medoids to form clusters. After the clusters are formed, the BS collects data at regular intervals. Generally, the normal node transmits the monitored data directly to CH if the CH is in its one-hop transmission range. Otherwise, it will choose a relay node to forward its data package to CH by greedy algorithm. In a greedy algorithm, node chooses a neighbor which is closer to the sink compared to itself as the relay node. After the data is received by CH, the CH compresses the data and forwards it to the BS. 

### 5.2. Clustering Results of Different Number of Sensors

**Figure 3:**
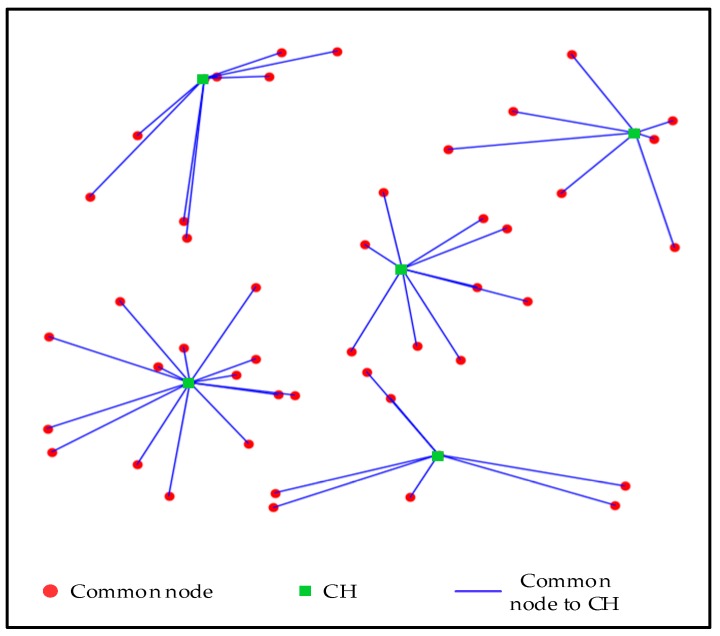
Cluster result of affinity propagation-based self-adaptive (APSA) algorithm (50 sensors).

Figure 3 shows the ultimate clustering result of APSA. As clearly shown in Figure 3, the proposed algorithm divides all the sensors into five clusters. The small dots denote the sensors, and the blue lines represent the virtual link between sensors and CHs.

**Figure 4:**
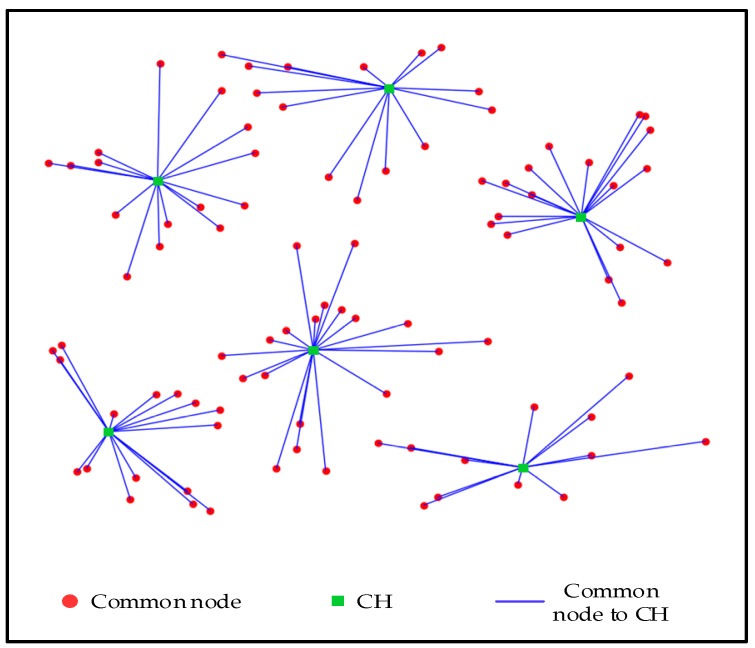
Cluster result of APSA (100 sensors).

Another 50 sensors are added to the network to test the presented algorithm and the simulation result is illustrated in Figure 4. APSA changes the number of CHs adaptively and it divides all sensors into six clusters. 

### 5.3. Analysis of Energy Consumption

**Figure 5:**
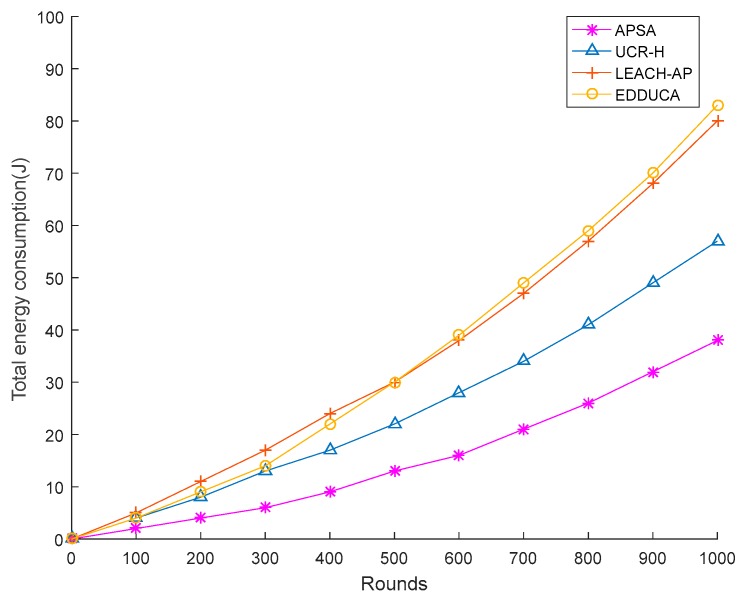
Energy consumption between different algorithms (50 sensor nodes).

The presented APSA is compared with LEACH-AP, UCR-H and EDDUCA which are all centralized routing protocols. For each protocol, 50 different samples of the network model are generated to execute the protocol and the result is based on the average value of repeated simulations. In Figure 5, the x-axis represents the number of rounds the network runs and the y-axis represents the total energy consumption of the network. It obviously shows that with rounds going, the total energy consumption of the presented APSA increases more slowly compared to the other three algorithms. In about the 1000th round, APSA achieves about 33.33%, 52.5% and 54.21% performance gain compared to UCR-H, LEACH-AP and EDDUCA respectively. 

### 5.4. Analysis of Network Lifetime

**Figure 6:**
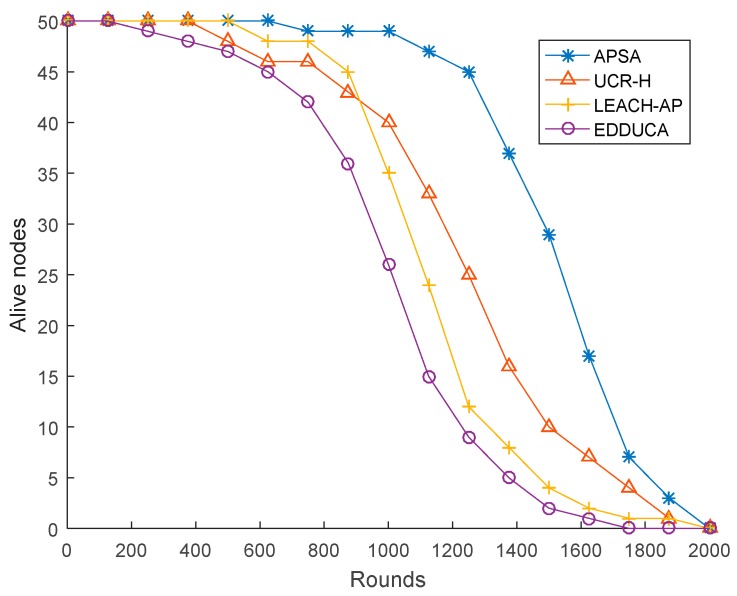
Alive nodes (50 sensor nodes).

The network lifetime is defined as the time when about half of the sensors in the network have dead. At this time, the network is divided into several isolated portions which leads to a serious decline in the performance of the network. In order to have a fair evaluation on different protocols, the same network model is used to execute the APSA, UCR-H, LEACH-AP and EDDUCA algorithms, respectively. The simulation result is demonstrated in Figure 6. As Figure 6 shows, the lifetime of APSA is 1511 rounds and it achieves about 16.23%, 31.39%, 51.1% performance gain compared to UCR-H, LEACH-AP and EDDUCA, respectively.

### 5.5. Analysis of Clustering Result

**Figure 7:**
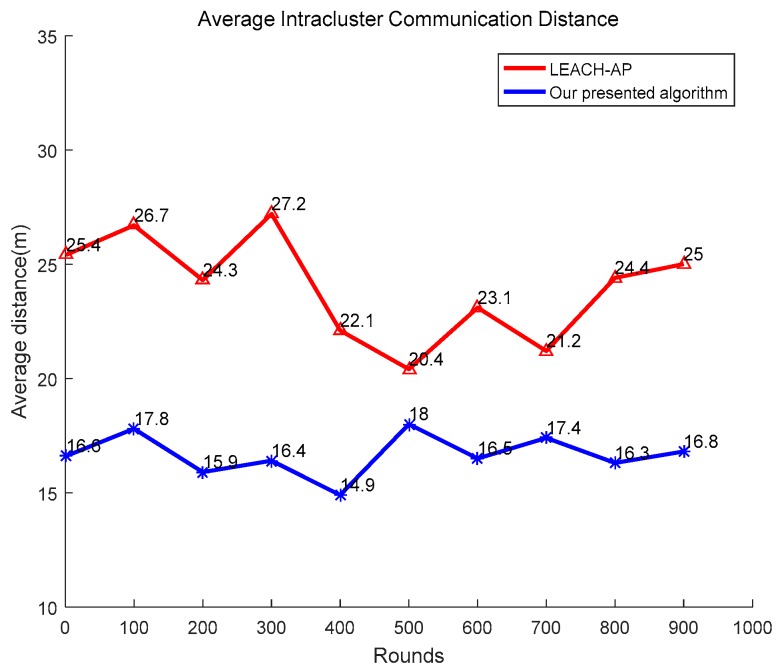
Comparison of average intracluster communication distance.

The reasonable CHs are expected to be selected during the selection procedure and one of the significant standards to evaluate the reasonability is the average communication distance between the CH and its members. The same simulation parameters are used and the number of sensors is set as 100. The presented algorithm is compared with LEACH-AP [19] and the simulation result is shown as Figure 7. From Figure 7, it can be seen that the presented APSA can greatly reduce the intracluster communication distance and improves about 30.5% performance compared to LEACH-AP.

**Figure 8:**
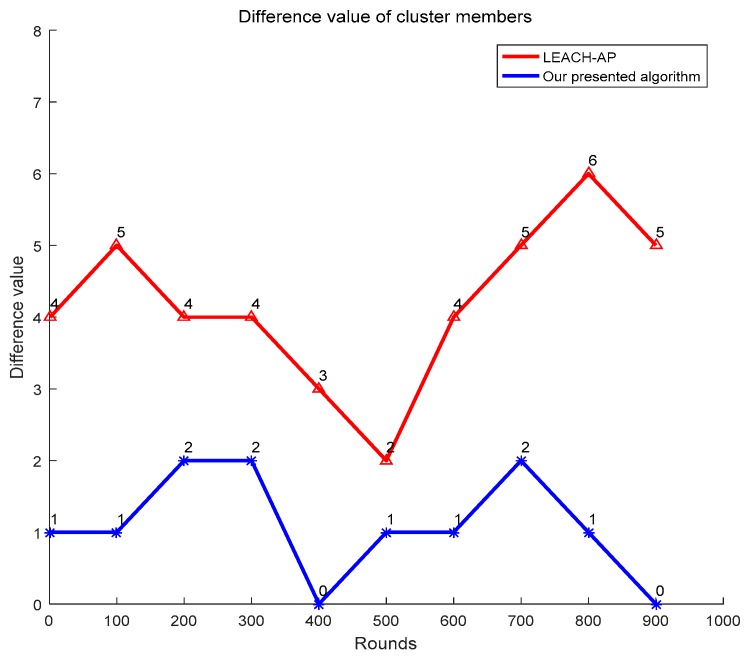
Comparison of difference values.

Uneven distribution of CHs will result in unbalanced energy consumption between clusters and accelerate the death of the node. The CHs distribute more evenly, the number of members for each cluster will be closer. Therefore, the even distribution of CHs can be evaluated by the difference value between the number of maximal and minimal cluster members. The simulation result is illustrated in Figure 8. From Figure 8, it can be seen that in the presented APSA, the difference value is 2 when the algorithm achieves the worst result; however, it can be 6 in LEACH-AP.

### 5.6. Study of Affinity Propagation (AP) Preference

The parameter p has a great impact on the performance of APSA in terms of convergence time and number of clusters. Different values of p are tested under the same network model (100 sensors) and the simulation result is shown as Table 3. As shown in Table 3, when p is set as –6000, the algorithm convergences faster and achieves a more reasonable number of clusters.

## 6. Discussion

The initial cluster centers are obtained by iteration using the AP algorithm. With the scale of the network increasing, the time used for calculation of initial cluster centers will also increase rapidly. Therefore, one drawback of the presented clustering method is that it is not suitable for WSNs on a large scale. Additionally, the presented APSA can be improved by adjusting the value of AP preference. The value of the AP preference is obtained by experience and it has a significant influence on the performance of the AP; –6000 is just a suitable value for AP preference and we cannot ensure it is the optimal value. Therefore, our future work will focus on optimizing the parameter P.

The simulator used in this paper is MATLAB and it can only simulate the real world. However, in real applications, many other problems need to be solved. For example, in the simulation environment, it is assumed that the transmissions between sensors are always successful; while in the real environment, transmission may fail due to the harsh environment or the busy communication channel. Therefore, the presented algorithm still needs to be improved to adapt to the real environment.

Our future work will mainly focus on the improvement of expandability of the method. We will also combine popular mobile sink technology as well as data fusion technology with our clustering method to further improve performance.

## 7. Conclusions

The design of an energy-efficient routing algorithm has always been an important research issue for WSNs. In this paper, an adaptive clustering method based on an AP algorithm is presented, which can reduce the average data transmission distance of the network and provide load balanced routing effect. It firstly introduces the AP algorithm to calculate the initial cluster centers. Then a modified K-medoids algorithm is adopted to partition the whole network into clusters according to the previous initial cluster centers calculated by AP. Simulation results show that about 33.33%, 52.5% and 54.21% performance gain can be achieved in terms of energy consumption, and about 16.23%, 31.39%, 51.1% performance gain can be achieved in terms of network lifetime compared to the UCR-H, LEACH-AP and EDDUCA algorithms respectively.

## Figures and Tables

**Table 1:** Comparison of routing protocols based on clustering.

Algorithm Name	Year	Structure	CH Election Features	Topology Control	Methods Used	Demerit
LEACH	2002	Two-layer structure	Random selection	Distributed		Uneven CH distribution
LEACH-C	2002	Two-layer structure	Residual energy, position	Centralized		High energy consumption
LEACH-AP	2016	Two-layer structure	position	Centralized	AP algorithm	Number of clusters assigning
PEGASIS	2002	Chain-structure	Position	Distributed	Greedy algorithm	Heavy network latency, poor robustness
HEED	2004	Two-layer structure	position	Distributed	Iteration	Long iteration time
TEEN	2001	Two-layer structure	Residual energy, position	Distributed	Iteration	Long iteration time
SECA	2012	Two-layer structure	Residual energy	Centralized	K-means algorithm	Unreason CHs selection
EAUC	2010	Two-layer structure	Residual energy, Position, number of neighbors	Centralized	Fuzzy logic system	High energy consumption
EEUC	2005	Two-layer structure	Residual energy, Position	Distributed	Iteration	High energy consumption
UCR-H	2017	Two-layer structure	Residual energy, Position	Centralized	Multiple CHs in each cluster	High energy consumption
EDDUCA	2016	Two-layer structure	Position	Centralized	Sierpinski triangle dividing	High energy consumption

**Table 2:** Simulation parameters.

Parameter	Definition	Value
*N*	Number of nodes	50
*coorBs*	Coordinate of the base station (BS)	(40,160)
*PS*	Packet Size for one communication	2000 bits
$E_{init}$	Initial energy of each node	2J
$E_{elec}$	Energy consumption per bit	50nJ/bit
$\varepsilon_{\mathrm{fs}}$	Transmitter amplifier (Free space model)	$10\mathrm{pJ}/\mathrm{bit}/m^{2}$
$\varepsilon_{\mathrm{mp}}$	Transmitter amplifier (Multi-path model)	$0.0013\mathrm{pJ}/\mathrm{bit}/m^{4}$
$E_{DA}$	Data aggregation energy	5nJ/bit/signal
*p*	Affinity propagation (AP) preference	−6000

**Table 3:** Results of different values of p.

**Value of p**	−4500	−5000	−5500	−6000	−6500	−7000	−7500
**Converge time (s)**	2.12	1.54	1.22	0.99	1.13	1.27	2.46
**Cluster number**	8	9	8	6	6	8	9

## Data Availability

The data that support the findings of this study are available from the corresponding author upon reasonable request.
